# Replication Dynamics for Six Gram-Negative Bacterial Species during Bloodstream Infection

**DOI:** 10.1128/mBio.01114-21

**Published:** 2021-07-06

**Authors:** Mark T. Anderson, Aric N. Brown, Ali Pirani, Sara N. Smith, Amanda L. Photenhauer, Yuang Sun, Evan S. Snitkin, Michael A. Bachman, Harry L. T. Mobley

**Affiliations:** a University of Michigan Medical School, Department of Microbiology and Immunology, Ann Arbor, Michigan, USA; b University of Michigan Medical School, Division of Infectious Diseases, Ann Arbor, Michigan, USA; c University of Michigan Medical School, Department of Pathology, Ann Arbor, Michigan, USA; Brigham and Women's Hospital/Harvard Medical School

**Keywords:** bacteremia, growth rate, bloodstream infection, generation time

## Abstract

Bloodstream infections (BSI) are a major public health burden due to high mortality rates and the cost of treatment. The impact of BSI is further compounded by a rise in antibiotic resistance among Gram-negative species associated with these infections. Escherichia coli, Serratia marcescens, Klebsiella pneumoniae, Enterobacter hormaechei, Citrobacter freundii, and Acinetobacter baumannii are all common causes of BSI, which can be recapitulated in a murine model. The objective of this study was to characterize infection kinetics and bacterial replication rates during bacteremia for these six pathogens to gain a better understanding of bacterial physiology during infection. Temporal observations of bacterial burdens of the tested species demonstrated varied abilities to establish colonization in the spleen, liver, or kidney. K. pneumoniae and S. marcescens expanded rapidly in the liver and kidney, respectively. Other organisms, such as C. freundii and E. hormaechei, were steadily cleared from all three target organs throughout the infection. *In situ* replication rates measured by whole-genome sequencing of bacterial DNA recovered from murine spleens demonstrated that each species was capable of sustained replication at 24 h postinfection, and several species demonstrated <60-min generation times. The relatively short generation times observed in the spleen were in contrast to an overall decrease in bacterial burden for some species, suggesting that the rate of immune-mediated clearance exceeded replication. Furthermore, bacterial generation times measured in the murine spleen approximated those measured during growth in human serum cultures. Together, these findings provide insight into the infection kinetics of six medically important species during bacteremia.

## INTRODUCTION

An estimated 11 million sepsis-associated deaths occur annually, accounting for nearly one in five deaths globally (2017), and sepsis is a principal cause of mortality among pediatric populations (1, 2). The severity of this condition is also reflected economically, as it is among the most expensive conditions treated in U.S. hospitals, costing $23.7 billion in 2013 (3). Infection of the bloodstream by bacteria, or bacteremia, is a leading cause of sepsis and can originate in both community and clinical settings (4–6). While precise accounting of these infections can be difficult to track due to inconsistencies in reporting methodologies, there are estimated to be up to 628,000 bloodstream infections (BSI) annually in the United States (7). In recent decades, the impact of bacteremia cases has been worsened by an increasing prevalence of antibiotic-resistant strains, which can confound treatment of these infections (8–10).

Gram-negative bacterial species account for a significant number of the reported BSI cases globally (ca. 40 to 50%) (4, 5, 9–11). Advances in molecular diagnostics have increased the ability of clinical laboratories to accurately assess which bacterial species are most frequently associated with BSI, although the prevalences of individual species vary considerably between study cohorts and geographic regions. Escherichia coli and Klebsiella pneumoniae are consistently among the most common etiologic Gram-negative pathogens (4, 5, 10–12). In addition, less-well-characterized species, such as Serratia marcescens, Citrobacter freundii, Enterobacter spp., and Acinetobacter baumannii, have been increasingly recognized as important causative agents of BSI and are associated with drug resistance phenotypes (12–14). To gain a better understanding of bacterial replication characteristics during BSI, we have chosen to investigate a six-organism cohort (Table 1), representing a cross-section of commonly encountered BSI pathogens from three bacterial families. These six species have a significant impact on the global public health burden, and the overarching goal of this work is to understand the physiological characteristics that make these organisms successful pathogens in the bloodstream environment.

**TABLE 1:** Bacterial strains used in this study

Species	Strain name	Isolation source	Reference(s)
Acinetobacter baumannii	AB0057	Human blood	50
Citrobacter freundii	UMH14	Human blood	19
Enterobacter hormaechei ^*a*^	UM-CRE_14	Endoscope, human respiratory	51
Escherichia coli	CFT073	Human blood	52, 53
Klebsiella pneumoniae	KPPR1	Rifampin-resistant derivative of ATCC 43816	54
Serratia marcescens	UMH9	Human blood	18

a UM-CRE_14 was originally described as Enterobacter cloacae but was designated *E. hormaechei* following additional sequencing (BioProject accession no. PRJNA401926) (V. J. DiRita, unpublished data).

Substantial progress has been made in characterizing the genetic requirements of *in vivo* fitness for the six species that are the focus of this study. In recent years, our group and others have utilized forward genetic screens to identify bacterial genes that contribute to the survival of E. coli (15–17), S. marcescens (18), C. freundii (19), and A. baumannii (20, 21) during systemic infection. In addition, the fitness requirements of E. coli (22, 23) and K. pneumoniae (24, 25) have been characterized in a urinary tract model and lung infection model, respectively, of note because the urinary tract and lung are exploited by these species for dissemination into the bloodstream. These studies together have identified numerous bacterial survival strategies during infection. However, the infection kinetics and replication dynamics of each species in the mammalian host remain less well characterized. The rapidly progressing nature of sepsis due to BSI makes investigation of bacterial replication an important component in the strategy to combat these infections.

The conventional means of determining bacterial infection dynamics, often by counting viable cells or using a reporter signal generated by the bacterial cells, are informative for identifying infection microenvironments and for determining the virulence capabilities of different strains. However, bacterial abundance at any point during infection is a result of the dynamic influences of immune-mediated clearance, dissemination, and bacterial replication rates. Assessments based on bacterial burden alone may also be confounding in the case of bacteremia since circulating organisms in the bloodstream may continually reseed local organs. Therefore, bacterial replication rates are an important component toward an understanding of fitness within the host environment. Several strategies have been utilized for measuring bacterial replication *in situ*, including plasmid segregation (26) and fluorescence microscopy (27). While also informative, these techniques require prior genetic manipulation of the organism in question. To minimize bacterial manipulation and streamline methodologies between our six species of interest, we optimized a genomic sequencing-based approach, termed the peak-to-trough ratio (PTR), to measure bacterial replication rates during infection (28). Sequence read quantification can be used to determine bacterial replication rates *in situ* by comparison with *in vitro* growth rate standards. This concept is based on the principle that the initiation of bacterial chromosomal replication occurs proportionally with generation time (*g*) (28). Under favorable growth conditions, where chromosomal replication time exceeds generation time, the progression of multiple simultaneous replication forks from the origin of replication results in an imbalance in chromosomal copy number relative to genome position. The resulting PTR is determined from the ratio of sequence coverage at the peak (replication origin) to the trough (replication terminus), when reads are mapped along the length of the bacterial genome. This principle has been used extensively to estimate the replication rates of bacterial populations from complex multispecies samples in both natural and experimental infections (28–32) as well as single-species infections in model systems (27, 33).

In this study, we have characterized the replication dynamics for E. coli, K. pneumoniae, S. marcescens, A. baumannii, C. freundii, and Enterobacter hormaechei in the mammalian host. A two-pronged approach, consisting of conventional bacterial burden measurements together with measurement of bacterial replication rates, demonstrates that these species are capable of robust replication during BSI and have unique kinetics of infection among the major organs of the bloodstream.

## RESULTS

### Bacterial burden in a murine model of Gram-negative BSI.

The systemic nature of BSI provides numerous local environments for bacteria to establish a replicative niche. Previous work has established that large numbers of bacterial cells reside in the spleens, livers, and kidneys of mice infected via the tail vein route (18, 19, 21, 23, 34, 35). To determine the relevant infection niches for each of our six species of interest, bacteria were introduced into the bloodstream of mice and the numbers of viable bacteria in the spleen, liver, and kidneys were tracked over time. It should be noted that robust infection with A. baumannii AB0057 in this model requires the establishment of an immunocompromised host state (21, 23), and throughout this work, A. baumannii-infected mice were pretreated with a monoclonal antibody that targets the myeloid cell lineage.

**FIG 1:**
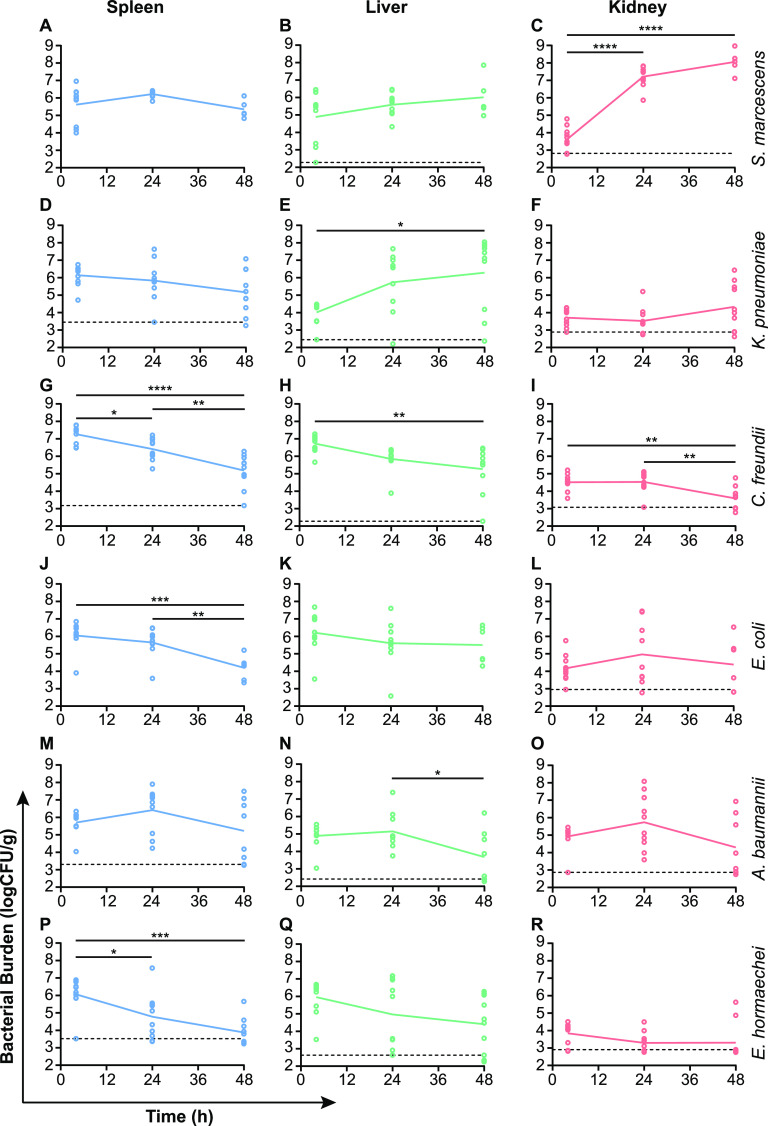
Tissue colonization for six bacterial species in a murine BSI model. C57BL/6J mice were inoculated (*n* = 10/time point) with S. marcescens (A to C), K. pneumoniae (D to F), C. freundii (G to I), E. coli (J to L), A. baumannii (M to O), or *E. hormaechei* (P to R) via tail vein injection. Target doses for each species were as follows: for A. baumannii, 1 × 10^7^ CFU; for C. freundii, 5 × 10^7^ CFU; for E. coli, 5 × 10^6^ CFU; for *E. hormaechei*, 1 × 10^8^ CFU; for K. pneumoniae, 5 × 10^5^ CFU; and for S. marcescens, 5 × 10^6^ CFU. Bacterial loads in the indicated organs were monitored by viable counts for 48 h. Time points are connected by the mean infectious burden, and horizontal dashed lines indicate the limits of detection, where applicable. Each graph reports the combined results from two independent experiments. Values were log transformed, and significant differences (*, *P* < 0.05; **, *P* < 0.01; ***, *P* < 0.001; ****, *P* < 0.0001) in mean bacterial burdens were assessed by one-way analysis of variance (ANOVA) with Tukey’s multiple-comparison test.

In some cases, the range of CFU recovered for a given bacterial species was greater at 24 and 48 h than at 4 h, suggesting that individual animals may have differences in their abilities to clear the bacterial infection despite our use of inbred mice (Fig. 1). For most species, the peak infectious burden in the spleen occurred immediately following infection, with total bacterial numbers decreasing over the subsequent 48-h period (Fig. 1). Although not statistically significant, A. baumannii and S. marcescens represented an exception to this trend in that the bacterial burden was observed to peak at 24 h, followed by a loss of viable cells by 48 h postinfection (Fig. 1A and M). The kinetics of bacterial burden in the murine liver were largely similar to that of the spleen for most of the tested species. K. pneumoniae was the only species that exhibited a significant increase in infectious burden in the liver during the time course (Fig. 1E), resulting in 190-fold more bacteria at 48 h than at 4 h and suggesting that K. pneumoniae is particularly well suited to colonizing the liver environment. Conversely, both C. freundii and A. baumannii exhibited significant decreases in liver bacterial burden (Fig. 1H and N), while *E. hormaechei* and E. coli also trended toward liver clearance (Fig. 1K and Q). Acute pyelonephritis due to hematogenous spread of bacteria to the kidneys is relatively uncommon, and the size of the inoculum used in the murine model may have contributed to the low level of recovery of most species observed here. However, S. marcescens proved to be a notable exception to this trend and exhibited a mean increase in bacterial burden in the kidneys by over 4,000-fold within 24 h (Fig. 1C). The rapid proliferation of S. marcescens in the kidneys suggests either an enhanced growth rate in this environment or a lack of clearance and was not matched even by the human pyelonephritis isolate E. coli CFT073 (Fig. 1L). The identification of species-specific niches and infection kinetics is expected given that each organism harbors a unique battery of fitness determinants and metabolic capabilities. However, these data provide only a partial assessment of the overall fitness of each species during BSI, and we sought to determine the *in situ* replication rates of each organism to better understand these infections.

### Establishing bacterial growth rates using genomic sequencing.

**FIG 2:**
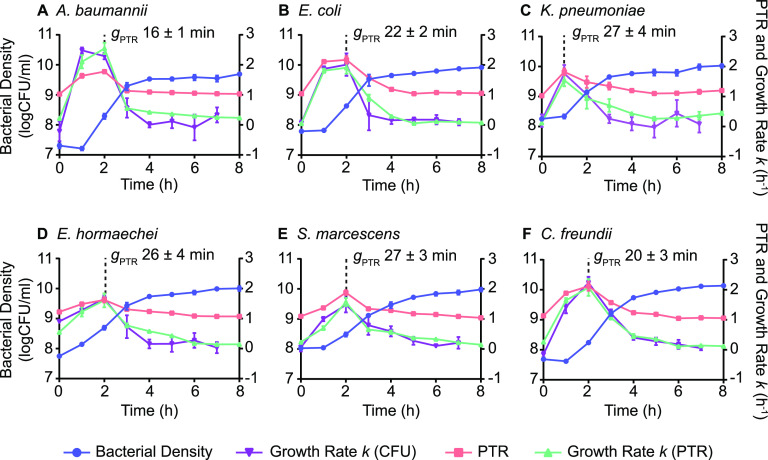
*In vitro* growth rate standards. Bacterial replication in Terrific broth was measured by viable counts in conjunction with whole-genome sequencing and PTR determination. Bacterial growth rate constants (*k*) (growth rate [CFU]) were determined based on the change in bacterial density at 1-h intervals, with the rate plotted at the starting point of each interval. PTR-based growth rate constants (*k*) (growth rate [PTR]) were determined by linear regression analysis calculated from the correlation between the viable count growth rate and the PTR (see Fig. 4). The mean generation time determined from PTR (*g*_PTR_; ±SD) at the peak growth rate for each species is indicated by the dotted line. Error bars represent the standard deviations from the means from three biological replicates.

**FIG 3:**
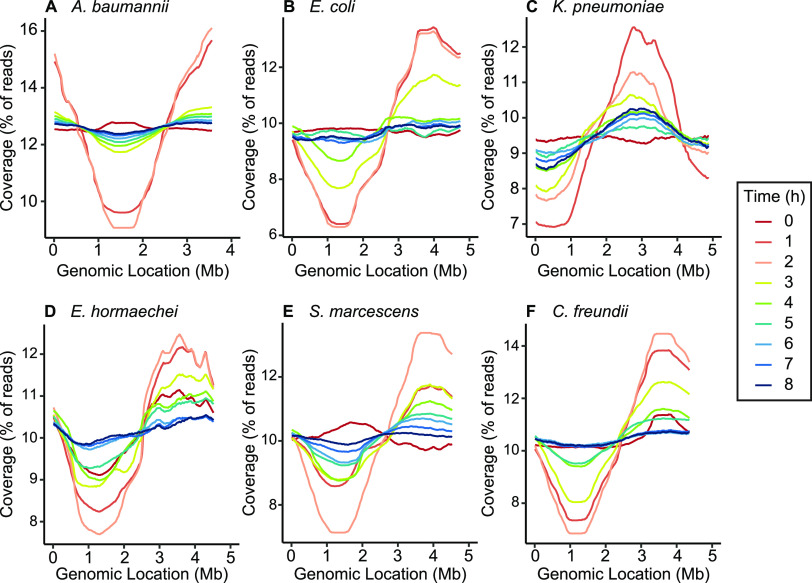
Read coverage as a function of chromosomal position during laboratory culture. Bacterial species were cultured in Terrific broth medium, and culture aliquots were collected hourly for isolation of genomic DNA and sequencing. PTR is defined as the ratio between the highest (peak) and the lowest (trough) read coverages. Lines indicate the mean coverage from three biological replicates.

**FIG 4:**
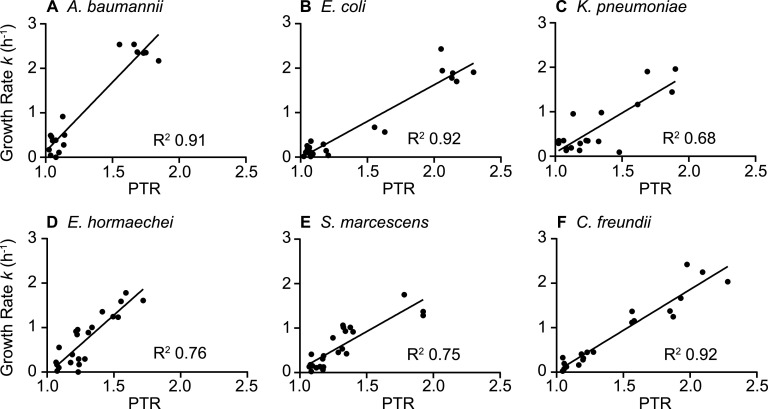
Linear relationship between PTR and growth rate. Growth rates constants (*k*) were determined hourly by viable counts in Terrific broth over an 8-h incubation period for the six bacterial species indicated. PTR was also determined by whole-genome sequencing for each sample. Correlations from three biological replicates throughout the time course were plotted excluding any negative *k* values that occurred during stationary phase. Linear regressions are represented by the solid lines.

To determine bacterial growth rates of the six selected species during bacteremia, it was first necessary to correlate conventionally determined growth rates with PTR values from mapped genomic sequence reads. Each of the six species was cultured in nutrient-rich Terrific broth to achieve a broad range of growth rates throughout the incubation period. Aliquots from *in vitro* cultures were collected in 1-h increments for sequencing and viable counts (Fig. 2). Plotting of total genomic sequence reads as a function of chromosomal position demonstrates that the ratio of sequence reads mapping to the origin of replication and replication terminus fluctuated throughout the growth curve (Fig. 3). The PTR calculated from the sequence read distributions modulated with the phases of bacterial growth, as measured from viable counts and with conventionally determined growth rates at 1-h intervals (Fig. 2). For each species, the highest PTR corresponds to the steepest portions of the bacterial density curve (log phase), confirming the previously established relationship between the PTR and growth rate (28). To quantify the relationship between the PTR and viable count growth rates for our specific bacterial strains, replicate values at each time point were plotted for all six species, and the linear relationship between these variables was determined (Fig. 4). The PTR-based growth rates were then calculated using these correlations and plotted alongside the viable count growth rates from which they were derived (Fig. 2). Bacterial generation times based on the PTR (*g*_PTR_) were also calculated. The peak *g*_PTR_ in laboratory medium occurred at 2 h postinoculation for all species except K. pneumoniae and ranged from 16 min (A. baumannii [Fig. 2A]) to 27 min (K. pneumoniae [Fig. 2C], S. marcescens [Fig. 2E]). The generation times derived from the PTR were consistent with the generation times determined from viable counts (*g*_CFU_) in the growth period between 1 and 3 h postinoculation (see Fig. S1 in the supplemental material), as was expected given the previously established linear relationship between these measurement methods. These strain-specific calibration curves of the PTR- and CFU-calculated growth rates were used for all subsequent experiments and, importantly, provide a means of assessing bacterial growth rates *in situ*.

10.1128/mBio.01114-21.1 FIG S1 Bacterial generation times in Terrific broth. Replication in Terrific broth was measured by viable counts. Generation times (*g*_CFU_) during the 2-h window of exponential-phase growth (shaded) were determined based on numbers of CFU. Bacterial density was determined from the mean of results from three biological replicates ± the standard deviation. Download FIG S1, EPS file, 1.2 MB. Copyright © 2021 Anderson et al. 2021 Anderson et al. https://creativecommons.org/licenses/by/4.0/ This content is distributed under the terms of the Creative Commons Attribution 4.0 International license.

As a test for the ability of this method to appropriately weigh any subpopulations having heterogeneous growth rates within a single sample, as may occur within host tissues during infection, existing reads from Terrific broth cultures representing rapidly and slowly replicating populations were computationally mixed in three different proportions. PTR values determined *de novo* from these mixed reads corresponded well with weighted PTR values that were projected from the original cultures of E. coli and S. marcescens (Table 2), demonstrating that this approach can accommodate disproportionate growth rates within a single sample.

**TABLE 2:** PTRs of mixed sequence reads from Terrific broth cultures

Species	% of sequence reads (PTR)		PTR of mixed reads	
	Fast	Slow	Expected^*a*^	Determined
E. coli	10 (2.296)	90 (1.207)	1.316	1.235
	50 (2.296)	50 (1.207)	1.751	1.525
	90 (2.296)	10 (1.207)	2.187	2.110

S. marcescens	10 (1.341)	90 (1.124)	1.145	1.123
	50 (1.341)	50 (1.124)	1.232	1.179
	90 (1.341)	10 (1.124)	1.319	1.298

a Weighted PTR values were calculated from the indicated proportions and original PTR of each population.

Since the recovery of bacterial DNA during infection was expected to be complicated by the presence of substantial amounts of host genetic material, it was also necessary to determine the sequencing depth at which reliable PTR determinations could be made. Random down-sampling of culture-generated sequence reads to model reduced genome coverage resulted in good fidelity of the PTR determinations relative to the original coverage plots, except with the lowest tested coverage level (0.01×) (Fig. S2). To assess the distribution of the change in the PTR as a function of coverage depth, ΔPTR values were plotted for each point along the growth curve (Fig. S3). Based on this analysis, the minimum coverage depth was set to 0.1×. The impact of subsampling to this level was further assessed by determining the correlation between the *g*_PTR_ at 0.1× and the original coverage. Subsampling to 0.1× coverage resulted in minimal loss of fidelity for generation times between ca. 10 and 100 min for each of the six species (Fig. S4), demonstrating reliable low-coverage growth rate determinations within this window. Longer generation times above 100 min, corresponding to the point of inoculation and stationary-phase growth, began to deviate from the original coverage data and therefore were not considered reliable at low coverage.

10.1128/mBio.01114-21.2 FIG S2 Determination of PTR at low sequence coverage. Mapped reads from Terrific broth cultures were down-sampled as described in Materials and Methods. The PTR was determined from the resulting sequence read distributions at each time point and compared to the original coverage level data. Each point represents the mean from four down-sampled replicates ± the standard error of the mean. Download FIG S2, EPS file, 1.6 MB. Copyright © 2021 Anderson et al. 2021 Anderson et al. https://creativecommons.org/licenses/by/4.0/ This content is distributed under the terms of the Creative Commons Attribution 4.0 International license.

10.1128/mBio.01114-21.3 FIG S3 Change in PTR resulting from subsample analysis. The change in PTR (ΔPTR, absolute value) after subsampling was compared to the original coverage PTR values for Terrific broth cultures. Subsampling was performed four times at each level, and individual values throughout the growth curve are shown. Black lines indicate the median and quartile levels. Download FIG S3, EPS file, 2.5 MB. Copyright © 2021 Anderson et al. 2021 Anderson et al. https://creativecommons.org/licenses/by/4.0/ This content is distributed under the terms of the Creative Commons Attribution 4.0 International license.

10.1128/mBio.01114-21.4 FIG S4 Correlation of generation times between 0.1× subsample and original coverages. Generation times (*g*_PTR_) were determined at the 0.1× minimum coverage subsample level (*n* = 4) and compared with the mean generation time (*n* = 3) at the original coverage level. The dotted line represents a hypothetical perfect correlation. Download FIG S4, EPS file, 2 MB. Copyright © 2021 Anderson et al. 2021 Anderson et al. https://creativecommons.org/licenses/by/4.0/ This content is distributed under the terms of the Creative Commons Attribution 4.0 International license.

### Replication rates in human serum.

**FIG 5:**
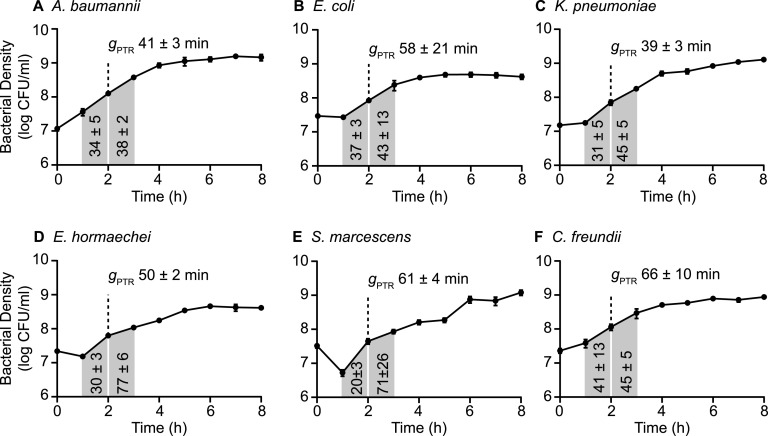
Bacterial replication in human serum. Each species was cultured in M9 base medium supplemented with 20% heat-inactivated human serum. Bacterial density was determined by viable counts for three biological replicates, and data points represent means ± SDs. Genomic DNA was isolated from bacteria at 2 h postinoculation, and whole-genome sequencing was performed to determine PTRs. The mean generation time (*g*_PTR_, ±SD) during exponential-phase growth is indicated for each species. Viable counts were used to calculate the mean *g*_CFU_ (shaded regions, ±SD) in minutes for the 1-h intervals bracketing the PTR collection point.

To measure bacterial replication rates under conditions that more closely approximate the bacteremia environment, the *g*_PTR_ was determined during exponential-phase growth for each of the six species cultured in M9 salts supplemented with 20% heat-inactivated human serum. It was expected that growth rates determined by CFU determination should again correlate with the rate calculated by the PTR, and indeed, the *g*_PTR_ values at 2 h postinoculation were largely within the range of *g*_CFU_ values that bracketed the PTR time points (Fig. 5). E. coli and C. freundii generation times exhibited higher degrees of variation in these experiments than the other four species; however, *g*_PTR_ values and the midpoint of the *g*_CFU_ values deviated by ≤1.5-fold in each case (Fig. 5B and F). These experiments also served to determine the levels of bacterial growth that can be achieved using only host-derived nutrient sources in the absence of complement-mediated killing, which may vary between the tested species. A. baumannii, *E. hormaechei*, and K. pneumoniae all achieved *g*_PTR_ of ≤50 min at midexponential phase under these conditions (Fig. 5A, C, and D), in contrast to the slowest generation time of 66 min, calculated for C. freundii (Fig. 5F). Not surprisingly, the generation times in diluted human serum were longer than those observed in an optimized laboratory medium (Fig. 2), reflecting an overall lower rate of growth. However, each of the six species was able to undergo substantial expansion using serum nutrients.

### Bacterial replication rates in the spleen following BSI.

The murine spleen was chosen as the target organ to determine bacterial generation times during infection due to the prominent role of this organ in blood homeostasis and the rapid accumulation of bacteria in the spleen following the introduction of bacteria into the bloodstream (34). Furthermore, the splenic bacterial burdens at 24 h postinfection were consistent among the tested species (Fig. 1). The generation time of S. marcescens in the kidney was also determined since the kinetics of infection in this environment represented a significant divergence from that observed in the spleen. Prior to the isolation of genomic DNA for sequencing, aliquots of organ homogenates from 24-h-infected mice were used to determine the bacterial burden (Table 3; Fig. S5). Total bacterial burdens for this series of infections were similar to those observed in Fig. 1 for all species. The remaining tissue homogenate was subjected to differential cell lysis to reduce the recovery of host genetic material, followed by sequencing and determination of PTRs (Fig. S6).

**TABLE 3:** Bacterial generation times in murine spleen and kidney^*a*^

Organism	Sample site	Bacterial burden (CFU/g)	Coverage	Pooled coverage	*g*_PTR_ (min)
A. baumannii	Spleen 1	1.2E+05	0.14×		48
	Spleen 2	2.2E+05	0.17×		116
	Spleen 3	8.4E+07	0.17×		30
	Spleen 4	1.1E+07	0.14×		25
	Spleen 5	1.8E+08	0.32×		34
E. coli	Spleen 1	1.1E+06	0.04×	0.19×	48
	Spleen 2	1.5E+06	0.05×		
	Spleen 3	7.5E+05	0.04×		
	Spleen 5	8.1E+04	0.05×		
	Spleen 4	5.3E+05	0.14×		44
K. pneumoniae	Spleen 1	6.2E+05	0.06×	0.23×	47
	Spleen 3	1.1E+06	0.09×		
	Spleen 4	1.6E+04	0.08×		
	Spleen 2	1.3E+04	0.11×		46
	Spleen 5	4.3E+03	0.25×		107
*E. hormaechei*	Spleen 1	5.9E+03	0.05×	0.25×	50
	Spleen 2	2.3E+05	0.09×		
	Spleen 3	1.5E+05	0.05×		
	Spleen 5	8.3E+04	0.06×		
	Spleen 4	3.8E+05	0.18×		44
C. freundii	Spleen 1	5.9E+04	0.20×		68
	Spleen 2	1.0E+05	0.20×		68
	Spleen 3	7.4E+06	0.18×		68
	Spleen 4	2.4E+09	0.81×		53
	Spleen 5	2.2E+05	0.08×		ND
S. marcescens	Spleen 1	1.8E+06	0.14×		57
	Spleen 2	2.6E+06	0.12×		55
	Spleen 3	2.7E+06	0.19×		54
	Spleen 4	2.9E+05	0.17×		67
	Spleen 5	3.1E+06	0.22×		44
	Kidney 1	1.2E+08	0.38×		84
	Kidney 2	7.9E+06	0.01×		ND
	Kidney 3	4.8E+07	0.21×		57
	Kidney 4	1.9E+05	<0.01×		ND
	Kidney 5	1.4E+08	0.78×		95

a Shading indicates pooled samples that did not individually meet the 0.1× coverage threshold. ND, not determined.

10.1128/mBio.01114-21.5 FIG S5 Murine infections for the determination of *in situ* growth rates. C57BL/6J mice were inoculated with the indicated bacterial species via tail vein injection. Mice were sacrificed 24 h later, and the bacterial burdens were determined by viable counts from homogenized spleen or kidneys. Bacterial DNA was isolated from the remaining homogenized tissue and sequenced for PTR determination. Horizontal lines represent the means (*n* = 5) of log-transformed bacterial burdens. Download FIG S5, EPS file, 0.9 MB. Copyright © 2021 Anderson et al. 2021 Anderson et al. https://creativecommons.org/licenses/by/4.0/ This content is distributed under the terms of the Creative Commons Attribution 4.0 International license.

10.1128/mBio.01114-21.6 FIG S6 Chromosomal read coverage in spleen and kidney samples. C57BL/6J mice were sacrificed 24 h after tail vein injection with the indicated bacterial species. Bacterial DNA isolated from spleen (A) and kidney (B) homogenates was sequenced, and reads were mapped to the genome of the infecting organism. The PTR was determined as the ratio between the maximum and the minimum coverage. Download FIG S6, EPS file, 2.2 MB. Copyright © 2021 Anderson et al. 2021 Anderson et al. https://creativecommons.org/licenses/by/4.0/ This content is distributed under the terms of the Creative Commons Attribution 4.0 International license.

The splenic bacterial *g*_PTR_ and genome coverages are shown in Table 3. All spleen samples from mice infected with A. baumannii and S. marcescens exceeded the coverage minimum of 0.1×, and the *g*_PTR_ was determined within individual spleens. For S. marcescens, the mean generation time in the spleen was 55 min (standard deviation [SD], ±8.0). The average generation time for A. baumannii was similar at 50 min (SD, ±37.7), though eliminating the spleen 2 outlier (*g*_PTR_, 116; Grubbs’ test α = 0.05) shortened the mean generation time considerably to 34 min (SD, ±9.8). There were four C. freundii-infected spleens from which independent *g*_PTR_ could be determined, resulting in a mean of 64 min (SD, ±7.6). The longer overall spleen generation times for C. freundii were consistent with the finding of slower growth for this organism in human serum than for the other tested species (Fig. 5). For infections that resulted in multiple samples below the 0.1× coverage minimum (E. coli, K. pneumoniae, and *E. hormaechei*), sequence reads from each set of spleens within a species cohort were pooled to determine PTRs (Table 3). Importantly, the pooled *g*_PTR_ for E. coli (48 min), K. pneumoniae (47 min), and *E. hormaechei* (50 min) were similar to those of individual replicates of each species that met the coverage threshold and demonstrated that these species were also capable of robust splenic replication. Together, these data demonstrate that each of the six tested species is well adapted for proliferation in the mammalian spleen following bloodstream inoculation, with most species exhibiting below 60-min generation times 24 h after initiation of infection and despite the numerous antibacterial strategies employed by the host. Furthermore, the overall trend toward decreasing numbers of CFU in the spleen beginning at 24 h postinfection (Fig. 1) suggests that the rate of immune clearance outpaces the substantial replication observed in this model.

The contrasting trajectories of S. marcescens burden in the spleen and kidneys (Fig. 1B and C) presented an opportunity to examine the impact of replication rates on bacterial accumulation in these two environments. The generation time of S. marcescens residing in the murine spleen (55 min) was not significantly different (Student’s *t* test, *P* ≥ 0.05) from that in the kidney (78 min; SD, ±19.7). In fact, the slightly longer generation times observed in the kidney demonstrate that the significant increase in bacterial abundance at this time point (Fig. 1C) was not due to an increased growth rate. Rather, a relative lack of immune-mediated clearance in the kidney compared to that in the spleen may facilitate the accumulation of S. marcescens at this site.

## DISCUSSION

There has been an abundance of important work characterizing the host response to the presence of bacteria and other microorganisms in the bloodstream, as well as the onset of sepsis due to dysfunction of the host immune system (36, 37). However, the replicative fate of bacteria following introduction to the bloodstream environment is unknown, and previous determinations of pathogen abundance in infection models represent only an endpoint of the combined influences of bacterial replication, cell death, hematogenous spread, and immune-mediated clearance. We establish here that six bacterial species responsible for the majority of Gram-negative BSI exhibit different infection kinetics, yet all are capable of robust replication in the murine spleen. For most species in most organs, the total bacterial burden decreases over time, yet the bacterial growth rates are rapid and similar to rates that are achieved during exponential growth in heat-inactivated human serum. Bacteria are therefore able to acquire adequate nutrients for efficient replication, but clearance by the host may exceed these replication rates. By determining both temporal bacterial viability and *in situ* replication rates, these findings together provide novel insight into the infection process that cannot be achieved by either measurement alone. Understanding the growth kinetics of bacteria during infection is important in designing treatment regimens, including the evaluation of antibiotic strategies, which in some cases require active growth to achieve bacterial killing. Furthermore, the evaluation of bacterial replication rates as a measurement of *in vivo* fitness will facilitate a future evaluation of individual gene function and their contribution to growth rate.

The mammalian bloodstream presents several challenges toward evaluating infection kinetics. Bacteria in circulation rapidly seed multiple organ systems, and to what extent ongoing tissue perfusion contributes to the bacterial burden of a given organ has not been explicitly determined for most species. Similarly, tissue-resident bacteria may act as a reservoir of infection and reenter the circulation, with the potential for spread to new foci. The low levels of bacteria isolated from blood itself in the murine model system and the modest impact of saline perfusion on the number of tissue-resident bacteria (34) suggest that the impact of circulating organisms on the total bacterial load is minimal. However, it remains possible that tissue-resident bacteria may have different growth characteristics from those of circulating bacteria. The measurements of *in vivo* bacterial replication rates presented here represent a population average, and to what extent subpopulations within a single organ may deviate from that average remains to be determined and would require a different experimental strategy.

Despite a recent adoption of sequence-based replication rate comparisons (29–31, 38, 39), literature reports of culture-standardized bacterial growth rates during infection or colonization remain limited. An advantage of the experimental infection approach taken here is that sequence reads recovered from tissue are mapped directly to the complete genomes of the specific infecting organism. This circumvents the additional considerations required when parsing reads from complex multispecies samples and performing *de novo* genome assembly. A similar approach has been utilized to determine that the rate of Staphylococcus aureus replication in human nasal samples is 2 to 4 h for the majority of individuals. The authors inferred from these findings that bacterial clearance rates were likely similar to replication rates in order to maintain stable colonization (32). Our group has also previously determined the rate of E. coli replication during urinary tract infection, establishing generation times of <40 min during early stages of experimental murine infection and even higher rates (17 to 34 min) for E. coli in the urine of women with active urinary tract infections (33). Importantly, both studies underscore the importance of the dynamic interaction between bacterial replication and clearance. The findings presented in this work also represent a baseline for bacterial growth rates during BSI. All six tested species were capable of generation times of less than 60 min in the spleen, with C. freundii exhibiting the slowest average time at 64 min. This continued replication in the presence of active clearance may be important for the onset of sepsis in that immune-stimulating products, such as lipopolysaccharide, are continually produced during bacterial growth.

Tracking tissue colonization during BSI reveals species-specific patterns of infection in our bacteremia model. For the majority of tested species, the highest bacterial burden in the spleen occurred immediately following infection. This is stark contrast to the much lower bacterial numbers initially observed in the liver of K. pneumoniae- and in the kidneys of S. marcescens-infected mice. Despite these relatively small founder populations, both instances resulted in the eventual expansion of bacteria by orders of magnitude. Interestingly, the increase of bacterial burden in the kidney by S. marcescens was not the result of an increased replication rate compared to that in the spleen. While it is possible that continued seeding of the kidney by circulating organisms contributes to some extent toward the observed proliferation of S. marcescens, a more likely explanation is that a lack of immune clearance of this species allows for unchecked expansion of S. marcescens in this environment. Assuming a constant generation time of 78 min, the mean time determined at 24 h postinfection (Table 3), we expect the kidney population of S. marcescens to undergo approximately 15 doublings between the 4-h and 24-h time points in Fig. 1C. Indeed, the S. marcescens kidney density observed at 24 h is achievable within 15 doublings from the 4-h starting density (data not shown). Unfortunately, it was not possible to determine the replication rate of K. pneumoniae in the murine liver due to technical limitations. The large liver mass compared to those of the spleen and kidney limited the efficiency of differential lysis and would have resulted in an insurmountable level of host DNA in sequencing samples without additional bacterial DNA enrichment. However, it is notable that K. pneumoniae was the only tested species to achieve a significant increase in population size in the liver during the infection time course. The K. pneumoniae strain used, KPPR1, encodes many of the hypervirulence-associated factors associated with liver abscesses and other infections in otherwise-healthy individuals (40). These include a hypermucoviscous capsule and additional iron transport systems (41), suggesting that both defense from host killing and efficient nutrient acquisition may contribute to K. pneumoniae expansion in this environment. Optimization of bacterial nucleic acid enrichment strategies may enable future studies to differentiate the contributions of these factors for liver-resident bacteria.

## MATERIALS AND METHODS

### Bacterial strains and culture conditions.

All bacterial strains used in this study have been previously described and are listed in Table 1. Routine culture of bacterial strains was conducted in LB medium (42). PTR growth rate standards were determined from bacteria cultured in Terrific broth (43). Bacterial growth in the presence of human serum was evaluated using M9 salts as the base medium (44) supplemented with 0.1 mM CaCl_2_, 1 mM MgSO_4_, and 20% heat-inactivated pooled human serum (Innovative Research).

### Determination of *in vitro* growth rates.

Bacterial strains were cultured overnight in LB medium, followed by inoculation at low density into prewarmed Terrific broth or M9 medium containing human serum. Viable bacteria from three biological replicates were enumerated by serial dilution and plating at 1-h intervals. Culture aliquots were also removed at the indicated time points, and harvested bacteria were frozen prior to isolation of genomic DNA and sequencing. The growth rate constant (*k*) was calculated using the standard equation *k* = ln(*N*_2_/*N*_1_)/(*t*_2_ − *t*_1_), where *N* is the density of viable bacteria (in CFU per milliliter) and *t* is time (in hours). Bacterial generation times (*g*) in minutes were determined using the standard formula *g* = (ln2/*k*) × 60.

### Murine model of bacteremia.

All experiments involving the use of laboratory animals were conducted with protocols approved by the University of Michigan Institutional Animal Care and Use Committee and were in accordance with Office of Laboratory Animal Welfare guidelines. Bacterial inocula were prepared from exponential-phase LB cultures that were washed with phosphate-buffered saline (PBS) prior to administration. Male and female C57BL/6J mice (6 to 8 weeks old) were infected via tail vein injection with 0.1-ml volumes of the bacterial suspensions (34). The administered dose was determined empirically for each organism and was based on obtaining reproducible infections with minimal moribund animals throughout the time course. Target doses for each species were as follows: for A. baumannii, 1 × 10^7^ CFU; for C. freundii, 5 × 10^7^ CFU; for E. coli, 5 × 10^6^ CFU; for *E. hormaechei*, 1 × 10^8^ CFU; for K. pneumoniae, 5 × 10^5^ CFU; and for S. marcescens, 5 × 10^6^ CFU. Animals that were to be infected with A. baumannii were rendered neutropenic by intraperitoneal injection with 500 ng of an anti-mouse Ly6G monoclonal antibody (Bio X Cell; clone 1A8) 24 h prior to infection. Following infection, mice were sacrificed at the indicated time points, and spleen, liver (excluding gallbladder), or kidneys were immediately homogenized in PBS on ice. Bacterial burdens were determined by serial dilution and plating of a small aliquot from each homogenate. Ten mice were infected for each time point in Fig. 1 over the course of two experiments. Homogenates from independent experiments used for PTR determination (*n* = 5/species) were further processed by differential cell lysis to reduce recovery of mouse DNA (33). Briefly, homogenates were diluted to 20 ml with PBS containing 1% Triton X-100 on ice. Following host cell lysis, bacteria were recovered by centrifugation at 4°C and washed once with PBS. Pellets containing recovered bacterial cells were stored at –80°C for isolation of genomic DNA.

### Isolation of genomic DNA and sequencing.

Genomic DNA for PTR determination was prepared using the DNeasy blood and tissue kit (Qiagen) or the Wizard Genomic DNA purification kit (Promega). All biological replicates were processed and sequenced independently. The generation of sequencing libraries, quality assessment, and genomic DNA sequencing were conducted by the University of Michigan Advanced Genomics Biomedical Research Core. Initial sequencing of all samples was performed using a HiSeq 2500 instrument (Illumina) with 50 single-end (SE) reads. Additional sequencing depth was obtained for *in situ* PTR samples using the same preparations of genomic DNA with NovaSeq (Illumina) and 50 paired-end (PE) reads.

### Read mapping and determination of PTR.

PTR values were determined using previously published methods (28, 33). Sequenced reads from each sample were cleaned and mapped to the complete genome sequence of the originating bacterial isolate (Table 1) using Trimmomatic v0.39 (45) and Bowtie2 v2.3.5 (–k 1, end-to-end) (46). Only reads with 100% identity to the appropriate bacterial reference genome were considered for the PTR. Kraken (47) was also used to verify the taxonomic origin of *in situ* sequence reads. S. marcescens kidneys 1 and 3 contained K. pneumoniae reads by this analysis but were not the result of coinfection. The contaminating K. pneumoniae reads did not map to the S. marcescens UMH9 genome or impact the PTR determinations for these samples. All alignments were indexed and sorted using SAMtools v1.11 (48), and the coverage depth for each nucleotide position was extracted using Bedtools v2.29.2 (49). A smoothing filter was applied to the mapped coverage of genomic segments, which was comprised of a moving sum with a window size of 10 kbp and a slide of 100 bp, followed by a moving median with a window size of 10-kbp bins and a slide of 100 bins. Instances where the bins did not have any mapped reads or had <50% remaining bins were discarded. The PTR was calculated from the peak and trough read coverage locations corresponding to maximum and minimum values, respectively, from the smoothed coverage (28). The minimum sequencing coverage required to accurately determine the PTR was estimated by down-sampling the *in vitro* Terrific broth reads from each species using seqtk v1.3 (https://github.com/lh3/seqtk). The original coverage levels, considering only the aligned sequence reads, were randomly subsampled to eight different coverage levels (0.01×, 0.05×, 0.1×, 0.3×, 0.5×, 1×, 5×, 10×). Each coverage threshold was sampled four times, each time by randomly subsampling reads using the seed levels 10, 15, 20, and 25. Sequence reads from individual spleen samples that did not meet the minimum genome coverage threshold were pooled and reanalyzed according to the schematic shown in Table 3. The pipeline can be accessed at https://github.com/alipirani88/Growth-rate-analysis.

### Sequence data availability.

Genomic DNA sequences have been deposited in the NCBI Sequence Read Archive and are available under accession number PRJNA657522.
